# The Battle between Retroviruses and APOBEC3 Genes: Its Past and Present

**DOI:** 10.3390/v13010124

**Published:** 2021-01-17

**Authors:** Keiya Uriu, Yusuke Kosugi, Jumpei Ito, Kei Sato

**Affiliations:** 1 Division of Systems Virology, Department of Infectious Disease Control, International Research Center for Infectious Diseases, Institute of Medical Science, The University of Tokyo, Tokyo 1088639, Japan; ngfkgwm@gmail.com (K.U.); jampei0513@yahoo.co.jp (J.I.); 2 Graduate School of Medicine, The University of Tokyo, Tokyo 1130033, Japan; 3 Laboratory of Systems Virology, Institute for Frontier Life and Medical Sciences, Kyoto University, Kyoto 6068507, Japan; kumoha12tsurumi@gmail.com; 4 Graduate School of Pharmaceutical Sciences, Kyoto University, Kyoto 6068501, Japan

**Keywords:** APOBEC3, lentivirus, Vif, arms race, gene diversification, coevolution

## Abstract

The APOBEC3 family of proteins in mammals consists of cellular cytosine deaminases and well-known restriction factors against retroviruses, including lentiviruses. *APOBEC3* genes are highly amplified and diversified in mammals, suggesting that their evolution and diversification have been driven by conflicts with ancient viruses. At present, lentiviruses, including HIV, the causative agent of AIDS, are known to encode a viral protein called Vif to overcome the antiviral effects of the APOBEC3 proteins of their hosts. Recent studies have revealed that the acquisition of an anti-APOBEC3 ability by lentiviruses is a key step in achieving successful cross-species transmission. Here, we summarize the current knowledge of the interplay between mammalian APOBEC3 proteins and viral infections and introduce a scenario of the coevolution of mammalian *APOBEC3* genes and viruses.

## 1. Introduction: Cross-Species Transmission of Pathogenic Viruses

In December 2019, an outbreak of unusual pneumonia, currently termed coronavirus disease 2019 (COVID-19), occurred in Wuhan, China. As of December 2020, COVID-19 has been an ongoing pandemic with more than 70 million cases and more than 1.5 million deaths worldwide [1]. A novel coronavirus, termed severe acute respiratory syndrome coronavirus 2 (SARS-CoV-2), was identified as the causative agent of COVID-19 [2,3]. Although it is assumed that SARS-CoV-2 was transmitted from bats to humans [4,5], the mechanisms by which SARS-CoV-2 was successfully transmitted into the human population are unknown.

Similar to SARS-CoV-2, many pathogenic viruses have been transmitted from animals to humans. The human immunodeficiency virus (HIV), the causative agent of acquired immune deficiency syndrome (AIDS), is a typical example of a virus that was transmitted from animals. HIV is a positive-sense, single-stranded RNA virus and belongs to the genus *Lentivirus* in the family Retroviridae [6]. Based on the viral genome sequence, HIV is classified into two viral groups: HIV type 1 (HIV-1) and type 2 (HIV-2). Previous studies have shown that HIV-1 is related to simian immunodeficiency virus (SIV) in chimpanzees (SIVcpz) [7,8], while HIV-2 is related to SIV in Old World monkeys and sooty mangabeys (SIVsm) [9]. These results suggest that HIV-1 and HIV-2, respectively, originated from cross-species transmissions of SIVcpz and SIVsm (more details are reviewed in references [10,11]).

**Figure 1:**
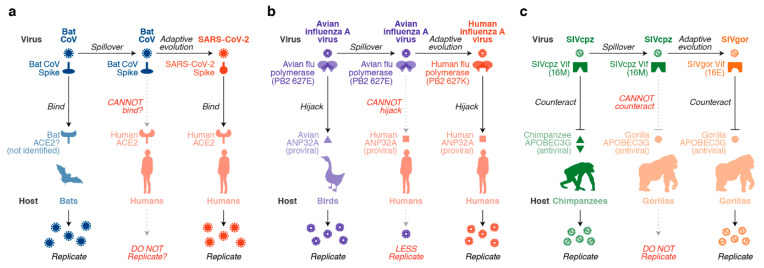
Association of host factors with viral cross-species transmission. (**a**) Usage of viral receptors for infection. In the case of SARS-CoV-2, its putative ancestral virus, a bat CoV, uses the receptor molecule(s) expressed on bat cells (not yet identified) (left). It is expected that a bat CoV was transferred to humans by a spillover event (although there may have been certain intermediate hosts between bats and humans). At first, the ancestral bat CoV may not have efficiently used human ACE2 as its receptor for infection (middle). However, through adaptive evolution, the bat CoV began to be able to efficiently utilize human ACE2 as the receptor and evolved into SARS-CoV-2 (right). (**b**) Hijacking of a proviral host protein. In the case of avian influenza A virus, viral proteases, particularly PB2, utilize avian ANP32A for efficient replication in avian cells (left). However, the avian flu polymerase cannot utilize human ANP32A (middle). Through adaptive evolution, the E627K mutation in the PB2 protein enabled the hijacking of human ANP32A for efficient replication in human cells (right). (**c**) Antagonism of an antiviral host protein. SIVcpz Vif can degrade and counteract the antiviral APOBEC3G protein of its host species (the chimpanzee) (left). In a spillover event, SIVcpz was transferred to gorillas. However, SIVcpz Vif cannot counteract the antiviral APOBEC3G protein of the new host (the gorilla) (middle). Through adaptive evolution, the virus acquired an M16E mutation in Vif, which enabled the virus to counteract gorilla APOBEC3G protein and efficiently replicate (right).

To accomplish cross-species transmission, viruses have to overcome certain “species barriers” in the new host (Figure 1). The switching of viral receptor tropism is one of the well-known examples of the mechanisms of actions leading to cross-species viral transmission. For instance, in the case of SARS-CoV-2, the amino acid sequence of the receptor binding domain of the spike protein is highly different from those of bat coronaviruses (CoVs), the putative origin(s) of SARS-CoV-2 [4]. Although the authentic origin of SARS-CoV-2 is not yet identified, it is hypothesized that its affinity for human angiotensin-converting enzyme 2 (hACE2) as the viral receptor is crucial for overcoming the species barrier and adapting to humans (Figure 1a) [4]. In addition to host proteins that are used as viral receptors for infection (e.g., human ACE2 for SARS-CoV-2), there are a variety of host factors that are potentially associated with the cross-species viral transmission. For instance, the viral polymerase (e.g., PB2) of avian influenza A virus acquires a single amino acid substitution (E627K) to utilize a mammalian host factor, acidic leucine-rich nuclear phosphoprotein 32 (ANP32A), for efficient viral replication in mammalian cells [12,13] (Figure 1b).

In addition to hijacking the proteins of the new hosts (e.g., ACE2 for SARS-CoV-2 and ANP32A for avian influenza A virus), viruses have to counteract the intrinsic antiviral proteins in the new hosts. Apolipoprotein B mRNA editing, catalytic polypeptide-like 3 (APOBEC3) family proteins, which are among the most investigated host factors, act as species barriers that potentially prevent the cross-species transmission of lentiviruses (reviewed in reference [10]). On the other hand, lentiviruses have acquired a viral factor, called viral infectivity factor (Vif), to overcome APOBEC3-mediated restriction (Figure 1c). Importantly, the interaction between the host APOBEC3 protein and the lentiviral Vif protein is strictly species-specific. Therefore, we can illuminate the scenario of virus–host coevolution by focusing on the interplay between APOBEC3 proteins and Vif.

In this review, we first briefly summarize the antiviral effects of APOBEC3 proteins and their antagonist Vif (Section 2). We then introduce the current understanding of the interplay between mammalian APOBEC3 proteins and viruses in the present (Section 3) and throughout evolution (Section 4). In Section 3, we introduce recent findings on the roles of the APOBEC3 proteins of mammals, particularly those of the great apes, in cross-species lentiviral transmission. In Section 4, we introduce recent knowledge of the evolution of *APOBEC3* genes in mammals.

## 2. Antiretroviral Effect of APOBEC3 Proteins and Its Antagonism by the Lentiviral Vif Protein

The APOBEC3 family of proteins consists of cellular cytosine deaminases that catalyze cytosine-to-uracil (C-to-U) substitutions. APOBEC3 proteins are members of the AID/APOBEC superfamily, the proteins of which commonly possess a zinc-dependent catalytic domain (Z domain) with the HxE/PCxxC motif. Previous studies have revealed that AID/APOBEC superfamily proteins are involved in immunity, metabolism, and infectious diseases (reviewed in [14,15]). In humans, there are seven APOBEC3 members (APOBEC3A, APOBEC3B, APOBEC3C, APOBEC3D, APOBEC3F, APOBEC3G, and APOBEC3H), and these seven genes are clustered in the locus sandwiched between the *CBX6* and *CBX7* genes on chromosome 22 [16,17]. In 2002, Sheehy et al. first identified APOBEC3G (formerly called CEM15) as a restriction factor that counteracts HIV-1 [18]. Subsequently, some other APOBEC3 proteins such as APOBEC3D, APOBEC3F, and APOBEC3H have been identified as anti-HIV-1 factors [19,20,21,22,23,24]. During the replication of retroviruses, including HIV-1, minus-stranded viral cDNA is synthesized by a viral reverse transcriptase (RT) using viral genomic RNA as the template. Subsequently, the synthesized viral DNA is integrated into the host genome as a provirus by a viral enzyme called integrase. To exhibit antiretroviral effects, APOBEC3 proteins are incorporated into virions and transferred into the newly infected cells. These APOBEC3 proteins target minus-stranded viral cDNA and induce C-to-U mutations. Consequently, G-to-A mutations are accumulated in the positive strand in the proviral sequence, which results in nonsense and/or missense mutations in viral genes. Additionally, the deaminase-independent inhibition of retroviral replication has also been reported: some APOBEC3 proteins directly bind to viral genomic RNA and/or minus-stranded cDNA and physically inhibit the elongation of reverse transcription [25,26,27]. In addition, APOBEC3G interacts directly with viral RT and blocks its function [28,29].

To counteract the antiviral activity of APOBEC3 proteins, lentiviruses have evolutionarily acquired an accessory gene, *Vif*, which was identified in the early 1980s and formerly called A [30], Q [31], P’ [32], ORF-1 [33], or sor (an abbreviation of short open reading frame) [34]. In 1987, Strebel et al. first demonstrated that *Vif* is an essential gene for HIV-1 infectivity in the A3.01 human CD4^+^ T cell line [35]. Then, in 2002, Sheehy et al. revealed that Vif degrades APOBEC3G in virus-producing cells and inhibits its incorporation into nascent viral particles [18]. Subsequent studies in this century have shown that the Vif protein interacts with APOBEC3 proteins as well as with the cellular components of the E3 ubiquitin ligase complex, which consists of Cullin 5 (CUL5), Elongin B/C (ELOB/C), RING-box protein 2 (RBX2), and core-binding factor subunit β (CBF-β), and induces the ubiquitination of substrate APOBEC3 proteins, leading to the degradation mediated by the 26S proteasome [36,37,38,39]. Interestingly, the requirement of CBF-β is specific for the Vif proteins of primate lentiviruses, and CBF-β is dispensable for the degradation of APOBEC3 proteins mediated by the Vif proteins of non-primate lentiviruses [40,41,42,43]. Instead of CBF-β, the Vif protein of Maedi-visna virus, a lineage of lentivirus infecting sheep, utilizes another cellular protein, cyclophilin A, for hijacking cellular E3 ubiquitin ligase complex, leading to the degradation of sheep APOBEC3 proteins [44]. These observations suggest that the evolutionary adaptation process of lentiviral Vif proteins is different among the lentivirus lineages.

## 3. Role of Mammalian APOBEC3 Proteins in Cross-Species Lentiviral Transmission

### 3.1. Great Ape APOBEC3 Proteins and Their Lentiviruses

Great ape lentiviruses were isolated from chimpanzees and gorillas but not from the other great apes such as the bonobos [45]. Based on phylogenetic analyses, HIV-1 is classified into four groups: M (major), N (non-M-non-O), O (outlier of outgroup), and P. HIV-1M and HIV-1N originated from SIVcpz, while the cross-species transmission of SIVgor (an SIV infecting gorilla) from gorillas to humans led to the emergence of HIV-1O and HIV-1P (in humans) [46,47,48,49]. Molecular phylogenetic analyses using the viral sequences obtained from HIV-1-infected individuals as well as from wild animals (e.g., chimpanzees and gorillas) in recent decades have enabled us to trace the evolutionary routes that led to the birth of HIV-1. However, the molecular mechanisms of the evolution and cross-species transmission of primate lentiviruses remain largely unclear. Elucidating the interplay between host factors and viral factors, including APOBEC3 proteins and Vif, can provide clues with which to understand such molecular mechanisms. There are at least two reports addressing the potential roles of primate APOBEC3 proteins as species barriers in the cross-species transmission of great ape lentiviruses. First, the Vif protein of SIVcpz, the ancestor of SIVgor, cannot counteract gorilla APOBEC3G, while SIVgor Vif can [49]. This observation suggests that gorilla APOBEC3G has played a role in restricting the cross-species transmission of SIVcpz from chimpanzees to gorillas as a species barrier and that SIVcpz Vif evolved into SIVgor Vif by acquiring the ability to counteract gorilla APOBEC3G. Intriguingly, Nakano et al. recently demonstrated that a single amino acid mutation (M16E) enabled SIVcpz Vif to degrade and counteract gorilla APOBEC3G [50]. Methionine (M) at amino acid position 16 is highly conserved in SIVcpz Vif, while glutamic acid (E) is conserved in SIVgor Vif [50]. Second, SIVcpz Vif proteins cannot counteract human antiviral APOBEC3H, while HIV-1M Vif can [51]. Because HIV-1M is the progeny of SIVcpz, this observation suggests that human APOBEC3H has potentially played a role in limiting the cross-species transmission of SIVcpz from chimpanzees to humans. Zhang et al. revealed that two amino-acid substitutions (EN47-48PH) permitted SIVcpz Vif to neutralize human APOBEC3H [51]. These observations suggest that great ape lentiviruses have evolved to adapt to new hosts by acquiring the ability to counteract antiviral factors in the new hosts. Moreover, these findings provide evidence suggesting that cross-species lentiviral transmission can be affected by antiviral APOBEC3 proteins in the new host.

### 3.2. Feline APOBEC3 Proteins and Their Lentiviruses

Evolutionary conflicts between APOBEC3 proteins and Vif can also be observed in non-primate mammals. For instance, feline APOBEC3 proteins can prevent the replication of feline lentiviruses, including feline immunodeficiency virus (FIV), while FIV Vif proteins can antagonize the antiviral activity of feline APOBEC3 proteins by degrading them [52,53,54]. Similar to primate APOBEC3 proteins, feline APOBEC3 proteins may work as species barriers for lentiviral transmission. In North America, puma lentiviruses (PLVs) have been isolated from two types of felids, namely pumas and bobcats. Based on their molecular phylogenies, these viruses are classified into PLV type A (PLV-A) and type B (PLV-B). Interestingly, PLV-A can be isolated from both pumas and bobcats, while PLV-B can be isolated only from pumas [55]. This observation suggests the possibility that bobcats intrinsically have species barrier(s) that restrict PLV-B infection. Konno et al. showed that puma and bobcat APOBEC3Z3 proteins (the orthologs of human APOBEC3H) can be antiviral [56]. Intriguingly, puma APOBEC3Z3 protein is degraded by both the PLV-A and PLV-B Vif proteins, while bobcat APOBEC3Z3 protein is degraded by PLV-A Vif but is resistant to PLV-B Vif [56]. These findings suggest that the cross-species transmission of PLV-B from pumas to bobcats can be hampered by bobcat APOBEC3Z3 protein. Furthermore, these findings strengthen the theory that APOBEC3 proteins work as a species barrier of lentiviral transmission in mammals.

On the other hand, an evolutionary pattern of lentiviral Vif and APOBEC3 proteins other than an evolutionary “arms race” has been observed in domestic cats (*Felis catus*). Based on its molecular phylogeny, the FIV that infects domestic cats (FIVfca) is classified into four subtypes: A, B, C and D [57,58,59]. Yoshikawa et al. showed that the Vif protein of FIVfca subtype B cannot degrade the two antiviral APOBEC3 proteins in domestic cats, APOBEC3Z3 and APOBEC3Z2Z3 [57]. Additionally, a molecular phylogenetic analysis has shown that FIVfca subtype B lost its ability to counteract these APOBEC3 proteins after divergence from the other FIVfca subtypes [57]. Furthermore, no Vif proteins of FIVfca subtype B tested (strains TM2, TM3, Aomori, Kyoto, and 2498B) counteracted the feline antiviral APOBEC3 proteins [57]. More intriguingly, previous reports have suggested that FIVfca subtype B is less pathogenic than the other subtypes and spreads worldwide [60,61]. Therefore, the inability of Vif to counteract APOBEC3 proteins implies that lentiviruses (at least for FIVfca subtype B) might be able to adapt to the host to attenuate their virulence.

## 4. Evolution of *APOBEC3* Genes in Mammals

### 4.1. Duplication and Diversification of APOBEC3 Genes

Human *APOBEC3* genes are encoded in a genetic locus sandwiched between the *CBX6* and *CBX7* genes (the “canonical locus” of *APOBEC3* genes) on chromosome 22 [16]. Notably, the numbers of *APOBEC3* genes differ among mammalian species. For example, humans have seven *APOBEC3* genes, while mice have only one, and marsupials have no *APOBEC3* orthologs [10,16,62]. Additionally, a recent study has reported that pteropid bats have 18 APOBEC3 Z domains in the canonical locus [63]. These findings suggest that the family of *APOBEC3* genes has been duplicated and diversified through mammalian evolution. Moreover, *APOBEC3* genes are under positive selection, and the amino-acid residues exposed on the protein surface, including the binding sites to the lentiviral Vif, are under particularly strong positive selection [62,64]. These observations raise the possibility that the evolution of *APOBEC3* genes has been driven by interaction with viral matter.

Recently, Ito, Gifford, and Sato hypothesized that the rapid evolution of mammalian *APOBEC3* genes has been driven by conflicts with ancient retroviruses and/or endogenous retroviruses (ERVs) [62]. ERVs are a lineage of retrotransposons and are the “genetic fossils” of ancient exogenous retroviruses because of their infection of germ cells [65]. The fact that ERVs occupy approximately 8% of the human genome [66] suggests there have been massive invasions of exogenous retroviruses into the genome of human ancestors in the past.

To depict the evolution and diversification of mammalian *APOBEC3* genes, Ito, Gifford, and Sato conducted a comparative genomic analysis of 160 mammalian species and identified 1420 *AID/APOBEC* family genes [62]. *APOBEC3* genes were found to be highly duplicated in some mammals, such as primates and bats, but other *AID/APOBEC* family genes were not [62]. By quantifying the number of ERV insertions in each mammalian genome and the level of accumulation of G-to-A mutations, which are the hallmarks of APOBEC3-mediated mutations in ERVs, it was demonstrated that the number of *APOBEC3* genes was significantly correlated with the number of ERVs and the frequency of G-to-A mutations in ERVs [62]. Furthermore, Ito, Gifford, and Sato examined the evolutionary timing of *APOBEC3* gene family expansion and the intensive genomic insertion of ERVs in primates and found that the times of these two events highly overlapped. These observations suggest that conflict with retroviruses was a major driving force in the evolution and amplification of *APOBEC3* genes in mammals.

### 4.2. The Birth of the APOBEC3G Gene

**Figure 2:**
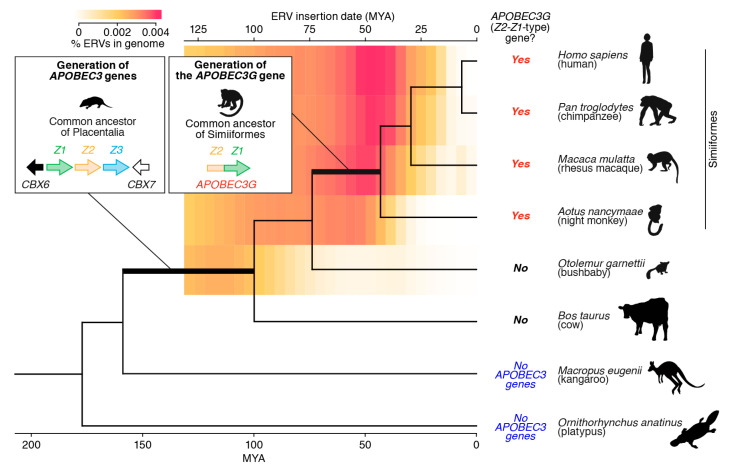
Birth of the *APOBEC3G* gene. Because the genomes of members of Monotremata (e.g., platypuses) and Marsupialia (e.g., kangaroos) do not encode *APOBEC3* genes in the canonical locus (i.e., the locus sandwiched between the *CBX6* and *CBX7* genes), it was previously assumed that *APOBEC3* genes were acquired in the common ancestor of Placentalia. Ito, Gifford, and Sato [62] revealed that the *APOBEC3G* gene (i.e., the Z2-Z1-type gene) is encoded in the genomes of Hominidae (e.g., humans and chimpanzees), Old World monkeys (e.g., rhesus macaques), and New World monkeys (e.g., night monkeys) but not in those of prosimians (e.g., bushbabies). These results suggest that the *APOBEC3G* gene (i.e., the Z2-Z1-type gene) was generated in the common ancestor of Simiiformes. Because the timing of the birth of the *APOBEC3G* gene overlapped with a period of high ERV invasion in the primate genome (shown as a heatmap) [62], it is speculated that retroviral invasions were a driving force in the generation of the *APOBEC3G* gene. MYA, million years ago.

As described above (Section 2), the *APOBEC3G* gene belongs to the *APOBEC3* family, which was first identified as a robust anti-HIV-1 restriction factor [18]. The gene structure of *APOBEC3G* contains two Z domains, Z2 and Z1, which are concatenated (i.e., they compose the Z2-Z1 domains) (Figure 2) [16]. Interestingly, the comprehensive genomic analysis by Ito, Gifford, and Sato [62] revealed that, in primates, the *APOBEC3* gene composed of Z2-Z1 domains is detectable in Simiiformes, which includes the great apes, Old World monkeys, and New World monkeys, but not in prosimians. These observations suggest that the *APOBEC3* gene composed of Z2-Z1 domains, namely, the *APOBEC3G* gene, emerged in the common ancestor of Simiiformes after diverging from prosimians (Figure 2). More intriguingly, ERVs massively invaded the genome of the common ancestor of Simiiformes approximately 50 million years ago [62]. Thus, the time frames of a massive ERV invasion and the birth of the *APOBEC3G* gene overlap in the common ancestor of Simiiformes. This provides strong evidence that the invasion of retroviruses and ERVs could be a driving force in the generation of the *APOBEC3G* gene.

### 4.3. Amplification of APOBEC3 Genes by Retrotransposition

Most mammalian *APOBEC3* genes are encoded in the canonical locus (reviewed in [10,16]), and amplifications of *APOBEC3* genes occur mainly within this locus [62,63]. These observations suggest that mammalian *APOBEC3* genes are amplified by tandem gene duplication. However, Ito, Gifford, and Sato [62] and Yang et al. [67] recently reported that some *APOBEC3*-like genes, particularly in New World monkeys, are encoded in genomic regions distinct from the canonical *APOBEC3* locus. These genes, encoded in non-canonical loci, have intronless structures and so are assumed to have been generated by the retrotransposition of *APOBEC3* genes from the canonical locus. Although most of these retrotransposed *APOBEC3G*-like genes (Yang et al. designated them “*APOBEC3G* retrocopies” [67]) are pseudogenized, some of them are intact [62,67]. Moreover, an *APOBEC3G* retrocopy was found to be conserved among all New World monkeys investigated [67]. Notably, the mRNA expression of *APOBEC3G* retrocopies has been detected in various tissues [62,67], which suggests the biological roles of *APOBEC3G* retrocopies in these animals. In fact, Yang et al. demonstrated that some of these retrocopies, particularly in marmosets and capuchins, decreased HIV-1 infectivity, while these genes did not affect the retrotransposition activity of LINE-1 [67]. Altogether, these findings suggest that *APOBEC3* genes can be amplified not only by tandem gene duplication but also by retrotransposition with retaining potential antiviral activity.

## 5. APOBEC3 Proteins and Non-Retroviruses

As described above, Vif is a well-studied viral protein that counteracts host APOBEC3 proteins. Other than Vif, some viral proteins such as HIV-1 protease [68] and RT [69], FIV protease [57], murine leukemia virus protease [70] and glycogag [71], human T-cell leukemia virus type I capsid [72], and mouse mammary tumor virus RT [73] potentially counteract host antiviral APOBEC3 proteins. These observations suggest that retroviruses have evolved a variety of strategies to counteract APOBEC3 proteins. In addition to retroviruses, it has been reported that human APOBEC3 proteins can potentially inhibit infections by human pathogenic non-lentiviruses such as hepatitis B virus (HBV) [74], human T-cell leukemia virus type I [72,75], human papillomavirus [76] and some human herpesviruses [77,78]. To counteract the antiviral effects of human APOBEC3 proteins, HBV utilizes a small nonstructural X (HBx) protein [74]. In contrast to lentiviral Vif (see Section 2), HBx does not induce the degradation of the APOBEC3G proteins expressed in HBV-infected hepatic cells [74]. Instead, HBx enhances the externalization of APOBEC3G protein via exosomes, resulting in decreases in intracellular APOBEC3G protein levels in infected cells [74]. In the case of herpesviruses, Cheng et al. demonstrated that human APOBEC3B protein is an antiviral against the Epstein–Barr virus (EBV), a human gammaherpesvirus, and notably that a viral protein called BORF2 counteracts the antiviral activity of the human APOBEC3B protein [77]. Interestingly, similar to HBV HBx in the case of the APOBEC3G protein, EBV BORF2 does not degrade the human APOBEC3B protein [77]. However, unlike HBV HBx, EBV BORF2 sequesters the human APOBEC3B protein from the nucleus, where EBV replicates [77]. Subsequent studies have further demonstrated that other human herpesviral proteins, such as ICP6 of the herpes simplex virus type 1 and ORF6 of Kaposi’s sarcoma-associated herpesvirus, exhibit similar activities against human APOBEC3A and APOBEC3B proteins [78,79]. Altogether, these observations suggest that mammalian *APOBEC3* genes have evolved to combat not only retroviruses (including lentiviruses) but also other pathogenic viruses, and that the respective viruses have sharpened their unique strategies to antagonize the activities of antiviral APOBEC3 proteins in order to support their own replication.

## 6. Conclusions

Here, we have summarized the current knowledge on the multiple aspects of mammalian *APOBEC3* genes, focusing particularly on their roles as species barriers that hamper cross-species transmission of lentiviruses (Section 3) and their evolutionary driving forces (Section 4). A variety of previous studies using biochemical, cell biological and molecular phylogenetic approaches have unveiled the functional and evolutionary relationships between lentiviral Vif proteins and the antiviral APOBEC3 proteins of the host species. The Vif–APOBEC3 interplay has been considered the consequence of an evolutionary “arms race” between lentiviruses and host mammals [10]. In other words, mammals have evolved antiviral genes, such as *APOBEC3* genes, to combat invasions of pathogenic viruses, while exogenous viruses have acquired viral genes, such as *Vif*, to counteract host antiviral genes for efficient replication.

In addition to the Vif–APOBEC3 interplay, we briefly summarized some examples of the interplay between the viral proteins encoded by non-lentiviral pathogenic viruses (e.g., HBV and some human herpesviruses) and antiviral APOBEC3 proteins (Section 5). It is intriguing that different viruses have acquired unique anti-APOBEC3 proteins (e.g., HBV HBx and EBV BORF2) that counteract APOBEC3 proteins in their own manner. On the other hand, the evolutionary scenario of FIVfca subtype B [57] is also interesting in another context: it seems that FIVfca subtype B has attenuated its Vif ability against antiviral APOBEC3 proteins of domestic cats to possibly become apathogenic (see also Section 3.2). In summary, the interactions between antiviral APOBEC3 proteins and their viral counteractants are more complicated than expected. Future investigations into APOBEC3 proteins and their viral counteractants can provide clues to elucidate the complex interactions and coevolution of viruses and host antiviral factors.

## Data Availability

The data are available at https://giffordlabcvr.github.io/A3-Evolution/.
